# Effects of Respite Care on the Quality of Life of Caregivers of Children With Autism Spectrum Disorder in Comparison With Parent Training: A Systematic Review and Meta-Analysis

**DOI:** 10.7759/cureus.101377

**Published:** 2026-01-12

**Authors:** Satoshi Takatani, Hisashi Nakaguchi, Junko Honda, Takafumi Soejima, Mari Kitao, Qiting Lin, Noriyuki Nishimura

**Affiliations:** 1 Graduate School of Health Sciences, Kobe University, Kobe, JPN; 2 Research Institute of Nursing Care for People and Community, University of Hyogo, Akashi, JPN; 3 Department of Family and Community Medicine, The Eighth Clinical Medical College of Guangzhou University of Chinese Medicine, Guangzhou, CHN; 4 Department of Family and Community Medicine, Foshan Hospital of Traditional Chinese Medicine, Foshan, CHN

**Keywords:** autism spectrum disorder, caregiver quality of life, meta-analysis, parent training, respite care, systematic review

## Abstract

Parents of children with autism spectrum disorder (ASD) experience substantial psychological, social, and physical burdens that negatively affect their quality of life (QoL). Although parent training (PT) is a well-recognized intervention for improving parental well-being, the effectiveness of respite care (RC) remains unclear. This systematic review and meta-analysis aimed to evaluate the effects of RC-containing programs in improving caregivers' QoL compared to PT programs. Six electronic databases were searched until September 2025 for quantitative studies that examined RC-containing or PT programs targeting caregivers of children with ASD aged 0-18 years. Eligible designs included randomized, quasi-randomized, pre-post, and cross-sectional studies. Outcomes were QoL measured using validated scales. The risk of bias was assessed using RoBANS-2. Random-effects meta-analyses were conducted using standardized mean differences (SMD). Five studies met the inclusion criteria for RC-containing programs, and 10 met those for PT programs. RC-containing programs showed a significant moderate effect on caregiver QoL (SMD = 0.45, 95% CI: 0.32-0.58; I² = 1%) with low heterogeneity (I² = 1%, τ² = 0.0057, p = 0.40). PT programs also demonstrated a significant but smaller effect (SMD = 0.31, 95% CI: 0.14-0.47; I² = 42%) with high heterogeneity (I² = 42%, τ² < 0.0001, p = 0.08). Although all included studies in both RC-containing and PT programs showed a high risk of bias, the point estimate suggested a comparative improvement in QoL among caregivers who received RC-containing support. RC-containing programs appear beneficial for improving the QoL of caregivers of children with ASD, and their effects are comparable to those of PT programs. This highlights the importance of recognizing RC as an essential component of family support and of integrating flexible, needs-based RC into clinical practice.

## Introduction and background

Autism spectrum disorder (ASD) is a neurodevelopmental disorder with an increasing prevalence worldwide. For instance, in the United States, the prevalence of ASD has increased approximately five-fold between 2000 and 2022, and current estimates suggest that 1 in 31 children has ASD [1]. ASD is characterized by impairments in social communication and restricted, repetitive patterns of behavior. These characteristics vary in severity and range across individuals but typically persist from early childhood into adulthood [2]. The recent transition from the Diagnostic and Statistical Manual of Mental Disorders, 5th ed. (DSM-5), to DSM-5-TR has emphasized the need for tailored support and intervention strategies. The DSM-5-TR provides criteria for assessing severity across social communication and restricted, repetitive behaviors, thereby facilitating more precise diagnoses and individualized treatment plans. This underscores the increasing recognition of the diverse challenges faced by parents of children with ASD [3].

As highlighted by the DSM-5-TR, the heightened stress associated with managing ASD-related behaviors and coordinating care significantly affects parental quality of life (QoL) [3]. Previous research that explored the impact of stress on the QoL of parents of children with ASD identified several specific stressors [4]. Parents of children with ASD experience heightened stress owing to communication difficulties, behavioral challenges, and the need for constant medical and educational interventions. This stress often leads to increased emotional and financial strain, resulting in diminished QoL [5]. It was also found that parental QoL scores were significantly and negatively correlated with parenting stress scores for both fathers and mothers of children with autism [6-8]. QoL is essential for understanding the experiences and challenges faced by children with ASD and their families. Furthermore, parental QoL is considered a factor that influences treatment outcomes for children with ASD, making research on the QoL of parents of children with ASD critically important [9,10].

The impact of the behavioral characteristics of children with ASD on their parents' QoL may be reduced by several coping strategies. Parents of children with ASD who received social support reported improved physical and emotional well-being [11-13]. Social support has also been associated with better QoL among caregivers of children with ASD [14]. Among the various social support services available to parents of children with ASD, respite care (RC) has attracted attention as a support strategy for parents of children with ASD. RC is gaining attention as a support strategy for parents of children with ASD. RC aims to provide temporary relief and refreshment for parents caring for children with ASD, with the expectation of reducing parental psychological stress [15]. Advances in medical technology have improved the survival rates of children with disabilities and complex healthcare needs, resulting in an increased burden of care within families and a corresponding increase in emotional, social, and economic stress. These changes have increased the demand for family support and social resources, and RC is regarded as an important support service for reducing the burden on these families and promoting the well-being of the entire family [16]. However, when parents of children with ASD access RC, they often have no choice but to rely on acute (A1) or primary care, which limits their use of RC, and its effectiveness has not been fully examined [17].

On the other hand, parent training (PT) is a well-recognized intervention targeting parents of children with ASD [18], with extensive reports on its effects on their QoL. PT is aimed at teaching parents of children with ASD strategies to manage their children’s behavior, enhance communication, and support the development of social skills. PT is time-limited (typically 10-20 sessions), and it emphasizes the role of parents as change agents [18]. Meta-analyses of PT have reported that it is effective in reducing problem behaviors in children with ASD and parental stress [19-21]. However, PT focuses on improving parenting skills, whereas RC focuses on providing respite from caregiving; the two approaches differ greatly in nature. To expand the range of intervention options, it is important to clarify the extent to which RC can have a unique effect on improving QoL by comparing (A2) these two approaches.

Previous studies have reported that RC is associated with reduced stress, depressive symptoms, and anxiety in caregivers of children with ASD [22-25]. Additionally, it has been reported that the use of RC can improve the QoL of parents of children with ASD by alleviating constant fatigue, fostering social interaction with friends, and promoting social participation [26]. However, some studies have reported that RC does not consistently or sustainably improve the well-being or stress levels of parents of children with ASD [27,28]. Furthermore, (A3) no meta-analysis has systematically examined the effects of RC on the QoL of parents of children with ASD.

This systematic review and meta-analysis aimed to evaluate the effectiveness of family support programs that incorporate RC elements (hereafter referred to as RC-containing programs) compared with PT programs in improving the QoL of parents of children with ASD.

## Review

Methodology

Information Sources

A systematic search of PubMed, CINAHL, Web of Science, Cochrane Central Register of Controlled Trials, PsycINFO, and Embase databases was conducted to identify relevant studies on "the effects of RC-containing programs on the QoL of parents with ASD children" and "the effects of PT programs on the QoL of parents with ASD children." The search was completed on September 12, 2025.

Search Strategy

Search terms for the effectiveness of RC-containing programs were as follows: "autism", "autistic disorder”, "autism spectrum disorder", "respite care", "unscheduled care", "short break", "short stay", "day care", and "day service". Meanwhile, studies on the effectiveness of PT programs were conducted using the following search terms: "autism", "autistic disorder", "autism spectrum disorder", "parent training", "self-help group", "peer support", "parent association", and "family association". There were no restrictions on date, language, or publication type for the search. Non-English articles were excluded during the subsequent screening process. EndNote versionX9 software (Thomson Reuters, New York, NY, USA) was used to manage the searched literature.

Inclusion and Exclusion Criteria

The inclusion and exclusion criteria for this review were set according to the Cochrane Handbook for Systematic Reviews of Interventions (version 6.5, 2024).

Participants

Participants were caregivers of 0- to 18-year-old children with ASD diagnosed by DSM-IV-TR, DSM-V, or DSM-V-TR. Children with an ASD-like condition or an underlying physical disorder and/or genetic abnormality were excluded.

Interventions

RC-containing programs: RC was defined as a type of break in which temporary care was provided to an individual with disabilities, with the primary goal of providing relief to the individual’s primary caregiver [16]. In this review, RC-containing programs included combined programs of RC and other methods, with the exception of PT programs.

PT programs: PT is the solid support for disruptive behavior in children with ASD, with the aim of reducing caregivers' daily life difficulties and learning to communicate with children with ASD for the caregivers [19]. In this review, PT programs included combined programs of PT and other methods, except for RC-containing programs.

Controls

In this review, controls included caregivers receiving standard care, except for RC-containing programs or PT programs.

Outcomes

The study outcome was the QoL of the caregivers measured by QoL scales for caregivers with children with ASD and the general population. QoL scales for mental health (stress, burden, etc.) were excluded.

Type of Studies

In this review, all study designs, including interventional (randomized controlled trials (RCTs) and non-RCTs) and observational study designs, were considered.

Study Selection

After the search, all identified articles were loaded into EndNote versionX9 software, and duplicates were removed. Two authors (ST and HN) independently screened all titles and abstracts against the inclusion criteria for the review. The full texts of the identified eligible studies were also evaluated in a similar manner by two independent reviewers. Studies that did not meet the inclusion criteria were excluded. Any disagreement was resolved through discussion or with the involvement of a third reviewer (NN) until consensus was reached.

Data Extraction

Data were extracted from the studies identified as eligible for inclusion in the review by two independent reviewers. The extracted data included details on the intervention content, target population, research methods, and outcomes relevant to the review question and research objectives.

Quality Assessment

This review was conducted in accordance with the PRISMA (Preferred Reporting Items for Systematic Reviews and Meta-Analyses) 2020 guidelines [29] to ensure the quality of the systematic review. The PRISMA 2020 checklist is shown in Table 1. The Revised Risk of Bias Assessment Tool for Nonrandomized Studies (RoBANS-2) was used to assess the quality of the included studies [30]. The RoBANS-2 consists of eight domains (comparability of the target group, target group selection, confounders, measurement of intervention/exposure, blinding of assessors, outcome assessment, incomplete outcome data, and selective outcome reporting). In each domain, the quality of included studies was rated as "high," "low," or "unclear." The overall risk of bias in each study yielded the worst risk of bias in any of the eight domains. Two authors (ST and JH) independently assessed each domain. Next, if the domains were rated differently, they were discussed until a consensus was reached. However, if no consensus could be reached, a third reviewer (NN) was consulted to determine the final rating of "high," "low," or "unclear."

**Table 1:** PRISMA 2020 checklist PRISMA: Preferred Reporting Items for Systematic Reviews and Meta-Analyses Source: [29]

Section and Topic	Item #	Checklist Item	Location Where Item Is Reported
TITLE			
Title	1	Identify the report as a systematic review.	Title page; Abstract title
ABSTRACT			
Abstract	2	See the PRISMA 2020 for Abstracts checklist.	Structured Abstract
INTRODUCTION			
Rationale	3	Describe the rationale for the review in the context of existing knowledge.	Introduction and Background, paragraphs 1–5
Objectives	4	Provide an explicit statement of the objective(s) or question(s) the review addresses.	Introduction and Background, final paragraph
METHODS			
Eligibility criteria	5	Specify the inclusion and exclusion criteria for the review and how studies were grouped for the syntheses.	Methodology: Inclusion and Exclusion criteria
Information sources	6	Specify all databases, registers, websites, organizations, reference lists and other sources searched or consulted to identify studies. Specify the date when each source was last searched or consulted.	Methodology: Information Sources
Search strategy	7	Present the full search strategies for all databases, registers, and websites, including any filters and limits used.	Methodology: Search Strategy
Selection process	8	Specify the methods used to decide whether a study met the inclusion criteria of the review, including how many reviewers screened each record and each report retrieved, whether they worked independently, and, if applicable, details of automation tools used in the process.	Methodology: Study Selection and Data Extraction
Data collection process	9	Specify the methods used to collect data from reports, including how many reviewers collected data from each report, whether they worked independently, any processes for obtaining or confirming data from study investigators, and, if applicable, details of automation tools used in the process.	Methodology: Study Selection and Data Extraction
Data items	10a	List and define all outcomes for which data were sought. Specify whether all results that were compatible with each outcome domain in each study were sought (e.g., for all measures, time points, analyses), and if not, the methods used to decide which results to collect.	Methodology: Inclusion and Exclusion criteria
	10b	List and define all other variables for which data were sought (e.g., participant and intervention characteristics, funding sources). Describe any assumptions made about any missing or unclear information.	Methodology: Inclusion and Exclusion criteria
Study risk of bias assessment	11	Specify the methods used to assess risk of bias in the included studies, including details of the tool(s) used, how many reviewers assessed each study and whether they worked independently, and, if applicable, details of automation tools used in the process.	Methodology: Quality Assessment
Effect measures	12	Specify for each outcome the effect measure(s) (e.g., risk ratio, mean difference) used in the synthesis or presentation of results.	Methodology: Statistical Analysis
Synthesis methods	13a	Describe the processes used to decide which studies were eligible for each synthesis (e.g., tabulating the study intervention characteristics and comparing against the planned groups for each synthesis (item #5)).	Methodology: Statistical Analysis
	13b	Describe any methods required to prepare the data for presentation or synthesis, such as handling of missing summary statistics or data conversions.	Methodology: Statistical Analysis
	13c	Describe any methods used to tabulate or visually display results of individual studies and syntheses.	Methodology: Statistical Analysis
	13d	Describe any methods used to synthesize results and provide a rationale for the choice(s). If meta-analysis was performed, describe the model(s), method(s) to identify the presence and extent of statistical heterogeneity, and software package(s) used.	Methodology: Statistical Analysis
	13e	Describe any methods used to explore possible causes of heterogeneity among study results (e.g., subgroup analysis, meta-regression).	Not performed (limited number of studies)
	13f	Describe any sensitivity analyses conducted to assess the robustness of the synthesized results.	Not performed (limited number of studies)
Reporting bias assessment	14	Describe any methods used to assess risk of bias due to missing results in a synthesis (arising from reporting biases).	Not performed (insufficient number of studies)
Certainty assessment	15	Describe any methods used to assess certainty (or confidence) in the body of evidence for an outcome.	Methodology: Data Extraction and Quality Assessment
RESULTS			
Study selection	16a	Describe the results of the search and selection process, from the number of records identified in the search to the number of studies included in the review, ideally using a flow diagram.	Results: Study Selection; Figures 1, 2 (PRISMA 2020 Flow Diagram)
	16b	Cite studies that might appear to meet the inclusion criteria, but which were excluded, and explain why they were excluded.	Results: Study Selection; Figures 1, 2 (PRISMA 2020 Flow Diagram)
Study characteristics	17	Cite each included study and present its characteristics.	Results: Characteristics of Included studies; Tables 2, 3
Risk of bias in studies	18	Present assessments of risk of bias for each included study.	Results: Meta-analysis
Results of individual studies	19	For all outcomes, present, for each study: (a) summary statistics for each group (where appropriate) and (b) an effect estimate and its precision (e.g. confidence/credible interval), ideally using structured tables or plots.	Results: Meta-analysis; Figures 3, 4 (Forest Plots)
Results of syntheses	20a	For each synthesis, briefly summarize the characteristics and risk of bias among contributing studies.	Results: Meta-analysis
	20b	Present results of all statistical syntheses conducted. If meta-analysis was done, present for each the summary estimate and its precision (e.g. confidence/credible interval) and measures of statistical heterogeneity. If comparing groups, describe the direction of the effect.	Results: Meta-analysis; Figures 3, 4 (Forest Plots)
	20c	Present results of all investigations of possible causes of heterogeneity among study results.	Results: Meta-analysis (I² reported)
	20d	Present results of all sensitivity analyses conducted to assess the robustness of the synthesized results.	Not applicable (not performed)
Reporting biases	21	Present assessments of risk of bias due to missing results (arising from reporting biases) for each synthesis assessed.	Not applicable (not performed)
Certainty of evidence	22	Present assessments of certainty (or confidence) in the body of evidence for each outcome assessed.	Results: Meta-analysis
DISCUSSION			
Discussion	23a	Provide a general interpretation of the results in the context of other evidence.	Discussion: 3rd & 4th paragraphs
	23b	Discuss any limitations of the evidence included in the review.	Discussion: 5th paragraph
	23c	Discuss any limitations of the review processes used.	Discussion: 5th paragraph
	23d	Discuss implications of the results for practice, policy, and future research.	Discussion: 2nd & 5th paragraphs
OTHER INFORMATION			
Registration and protocol	24a	Provide registration information for the review, including register name and registration number, or state that the review was not registered.	Not applicable (not performed)
	24b	Indicate where the review protocol can be accessed, or state that a protocol was not prepared.	Not applicable (not performed)
	24c	Describe and explain any amendments to information provided at registration or in the protocol.	Not applicable (not performed)
Support	25	Describe sources of financial or non-financial support for the review, and the role of the funders or sponsors in the review.	Ethics Statement and Conflict of Interest Disclosures: Payment/services info
Competing interests	26	Declare any competing interests of review authors.	Ethics Statement and Conflict of Interest Disclosures: Conflicts of Interest
Availability of data, code and other materials	27	Report which of the following are publicly available and where they can be found: template data collection forms; data extracted from included studies; data used for all analyses; analytic code; any other materials used in the review.	Acknowledgments

Statistical Analysis

Studies with similar characteristics were included in this meta-analysis. Effect sizes were calculated as standardized mean differences (SMDs) for continuous data when different instruments were used to measure outcomes, and relative effect estimates were assigned 95% confidence intervals (95% CI). The clinical features of caregivers of children with ASD are highly dependent on their cultural background, and the true effects are likely to differ across studies in different countries. In addition, because methodological heterogeneity was observed in the identified studies, we applied a random-effects model [31,32]. Standard chi-square and I² tests were used to assess statistical heterogeneity among the studies. Statistical analyses were performed using EZR version 1.35 [33].

Results

*Study Selection*

**Figure 1:**
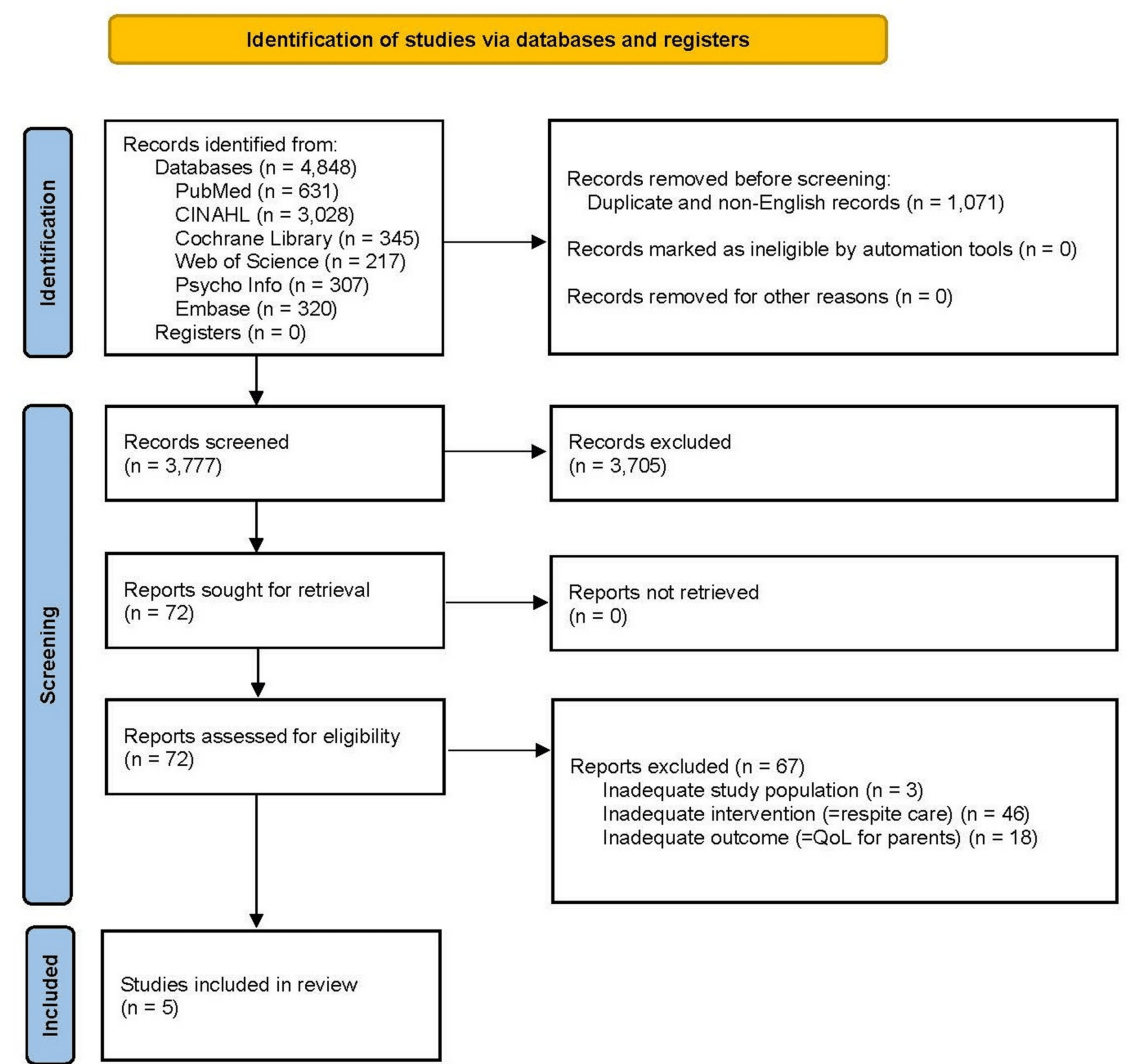
PRISMA 2020 flow diagram of the study selection process for RC-containing studies PRISMA: Preferred Reporting Items for Systematic Reviews and Meta-Analyses; QoL: quality of life; RC: respite care Source: [29]

**Figure 2:**
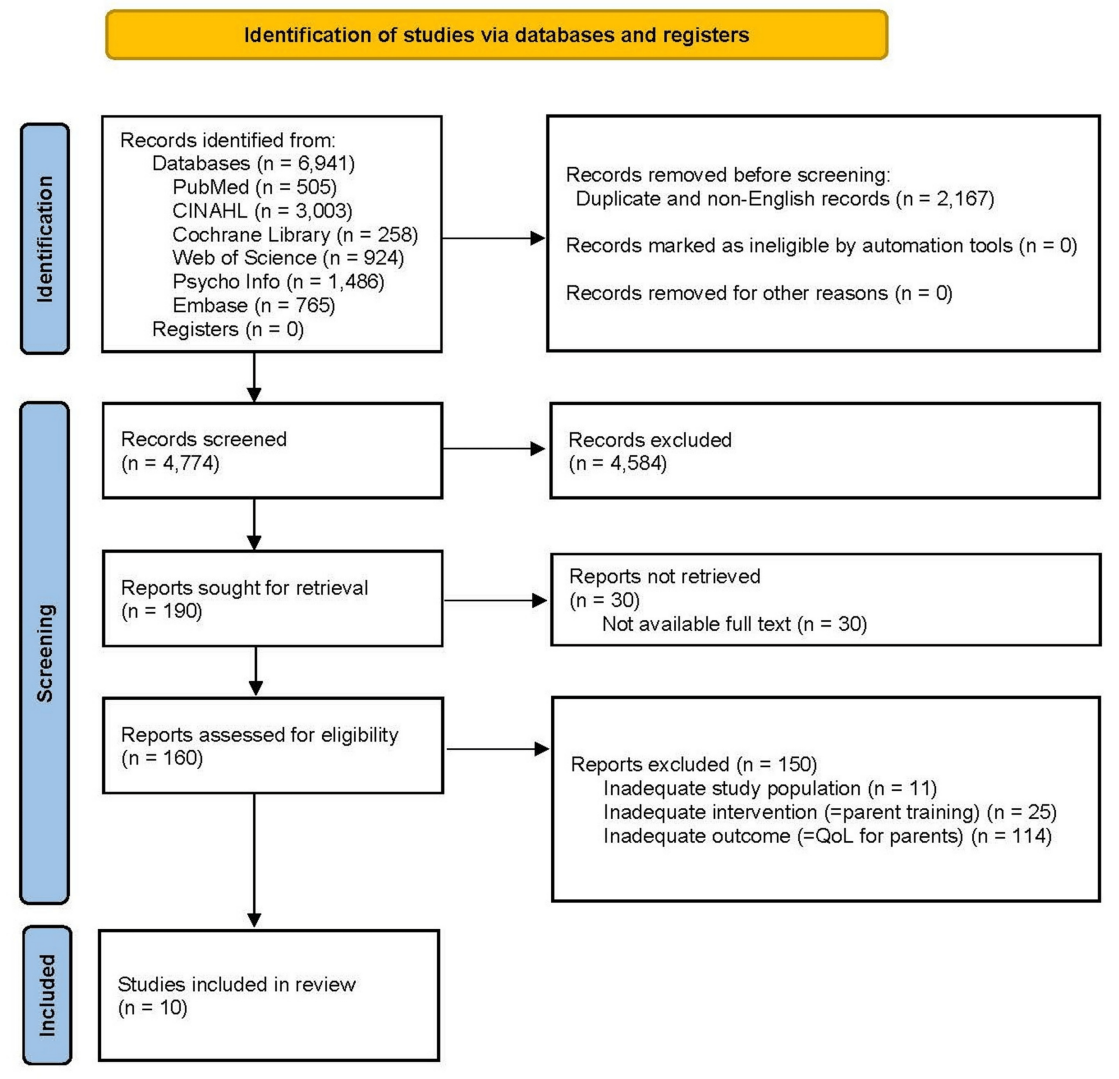
PRISMA 2020 flow diagram of the study selection process for PT studies PRISMA: Preferred Reporting Items for Systematic Reviews and Meta-Analyses; QoL: quality of life; PT, parent training Source: [29]

The study selection process is shown in Figures 1, 2 (PRISMA 2020 flow diagram).

Effects of RC-Containing Programs

From the database search, 4,848 articles were identified. Duplicates and non-English articles were excluded, leaving 3,777 articles. Subsequently, the full text of 72 papers screened by title and abstract was reviewed, and five studies met the eligibility criteria.

Effects of PT Programs

From the database search, 6,941 articles were identified. Duplicates and non-English articles were excluded, leaving 4,774 articles. Subsequently, the full text of 190 papers screened by title and abstract was reviewed, and 10 papers met the eligibility criteria.

Characteristics of the Included Studies

Tables 2, 3 present the characteristics of the included studies.

**Table 2:** Characteristics of included RC-containing studies NA: not available; SD: standard deviation; RC: respite care; QOL: quality of life; ASELCCs: Autism Specific Early Learning and Care Centres; HCBS: Home and Community-Based Services; MASS-R: Maryland Autism Services Survey-Revised; QOL-A: Quality of Life Autism; ROBANs-2: The Revised Risk of Bias Assessment Tool for Nonrandomized Studies Source: [30]

Author Name	Year	Country	Study Design	Participants / Controls	Age of Caregivers (Year) (Participants / Controls)	Age of Children (Year) (Participants / Controls)	Sampling Methods (Participants / Controls)	Intervention	Outcome Measure	Study Quality (RoBANS-2) [30]
SooHoo [26]	2019	USA	Non-randomized interventional study	16 caregivers (Pre-intervention) / 16 caregivers (Post-intervention)	Mean: 48.90 (SD= 10.10) / Mean: 48.90 (SD= 10.10)	Mean: 16.20 (SD= 6.37) / Mean: 16.20 (SD= 6.37)	Recruited from caregivers who had cruised with Autism on the Seas within the past 3 years / Recruited from families who had cruised with Autism on the Seas within the past 3 years	Autism on the Seas program	Beach Center Family QOL Scale	High risk of bias
											Eskow et al. [34]	2019	USA	Non-randomized interventional study	230 caregivers (Pre-intervention) / 230 caregivers (Post-intervention)	NA / NA	Mean: 15.91 (SD= 2.85) / Mean: 15.43 (SD= 2.87)	Recruited from Medicaid HCBS waiver families / Recruited from negligible waiver-like services registry families	Medicaid HCBS waiver programs	Beach Center Family QOL Scale	High risk of bias
Due et al. [35]	2018	Australia	Non-randomized interventional study	27 caregivers / 23 caregivers	Mean: 40.00 (SD= 6.3) / Mean: 35.10 (SD= 4.50)	Mean: 5.00 (SD= 2.00) / Mean: 3.90 (SD= 0.82)	Recruited from caregivers of children attending ASELCCs / Recruited from caregivers on the waitlist or recently commenced at ASELCCs	ASELCC services	QOL-A parent version	High risk of bias											
											Eskow et al. [36]	2015	USA	Non-randomized interventional study	130 caregivers (Pre-intervention) / 130 caregivers (Post-intervention)	NA / NA	Mean: 13.97 (SD= 3.31) / Mean: 13.16 (SD= 3.56)	Recruited from Medicaid HCBS waiver families. / Recruited from negligible waiver-like services registry families.	Medicaid HCBS waiver programs	Family QOL Scale (embedded in MASS-R)	High risk of bias
Eskow et al. [37]	2011	USA	Non-randomized interventional study	228 caregivers / 627 caregivers	Mean: 46.00 (SD= NA) / Mean: 42.60 (SD= NA)	Mean: 13.40 (SD= NA) / Mean: 9.53 (SD= NA)	Recruited from Medicaid HCBS waiver families / Recruited from negligible waiver-like services registry families (waiting list) for waiver services	Medicaid HCBS waiver programs	Beach Center Family QOL Scale	High risk of bias											

**Table 3:** Characteristics of included PT studies NA: not available; SD: standard deviation; PT: parent training; QOL: quality of life; ABC: Antecedent-Behavior-Consequence; ADI-R: Autism Diagnostic Interview-Revise; ASD: autism spectrum disorder; CARES: Counselling, Assistance, Reinforcement and Empowerment Services; CIDD: the Carolina Institute for Developmental Disabilities; FITT: Family Implemented TEACCH for Toddlers; IPAT: Integrative Parents’ Autism Training; M-CHAT-R/F: Modified Checklist for Autism in Toddlers-Revised with Follow-Up; PedsQL-FIM: Pediatric Quality of Life Inventory – Family Impact Module; QLESQ-SF: Quality of Life Enjoyment Satisfaction Questionnaire– Short Form; RAND-36: RAND 36-item health survey; SAAAC: South Asian Autism Awareness Centre; SF-16: short form 36 health survey; TEACCH: Treatment and Education of Autistic and related Communication-handicapped Children; UNC: University of North Carolina; WHOQOL 26: World Health Organization-Quality of Life 26; WHOQOL-BREF: World Health Organization Quality of Life-brief; RoBANS-2: The Revised Risk of Bias Assessment Tool for Nonrandomized Studies Source: [30]

Author Name	Year	Country	Study Design	Participants / Controls	Age of Caregivers (Year) (Participants / Controls)	Age of Children (Year) (Participants / Controls)	Sampling Methods (Participants / Controls)	Intervention	Outcome Measure	Study Quality (RoBANS-2) [30]
Qu et al. [38]	2024	China	Non-randomized interventional study	19 caregivers (Pre-intervention) / 19 caregivers (Post-intervention)	NA / NA	Mean: 3.25 (SD= 0.95) / Mean: 3.25 (SD= 0.95)	Recruited via a digital portal among caregivers of children with ASD who exceeded cutoff scores on the M-CHAT-R/F and ADI-R / Recruited via a digital portal among caregivers of children with ASD who exceeded cutoff scores on the M-CHAT-R/F and ADI-R.	A culturally adapted, group-based parent coaching program via telehealth	WHOQOL-BREF	High risk of bias
Mavroeidi et al. [39]	2024	Greece	Non-randomized interventional study	62 caregivers (Pre-intervention) / 62 caregivers (Post-intervention)	Mean: 42.80 (SD= 5.80) / Mean: 42.80 (SD= 5.80)	Mean: 9.20 (SD= 5.30) / Mean: 9.20 (SD= 5.30)	Recruited in Greece, Italy, Spain, and Turkey through autism-related institutions, family associations, schools, and local service providers (some via printed invitations) / Recruited in Greece, Italy, Spain, and Turkey through autism-related institutions, family associations, schools, and local service providers (some via printed invitations).	The Intervention: IPAT Training Activity Using the IPAT Module	WHOQOL-BREF	High risk of bias
Mills et al. [40]	2021	Canada	Non-randomized interventional study	63 caregivers (Pre-intervention) / 63 caregivers (Post-intervention)	Mean: 43.73 (SD= 8.85) / Mean: 43.73 (SD= 8.85)	Mean: 11.30 (SD= 7.02) / Mean: 11.30 (SD= 7.02)	Recruited from caregivers receiving or newly referred for services at the SAAAC Autism Centre (Toronto, Canada) / Recruited from caregivers receiving or newly referred for services at the SAAAC Autism Centre (Toronto, Canada).	The CARES program	QLESQ-SF	High risk of bias
Akhani et al. [41]	2021	Iran	Non-randomized interventional study	19 caregivers (Pre-intervention) / 19 caregivers (Post-intervention)	Mean: 37.05 (SD= 2.79) / Mean: 37.05 (SD= 2.79)	Range: 3-5 / Range: 3-5	Recruited from two public and three private clinics in Tehran (Iran) / Recruited from two public and three private clinics in Tehran (Iran).	Ingersoll’s parent training protocol	WHOQOL-BREF	High risk of bias
Turner-Brown et al. [42]	2019	USA	Non-randomized interventional study	32 caregivers (Pre-intervention) / 32 caregivers (Post-intervention)	NA / NA	Mean: 2.50 (SD= 0.40) / Mean: 2.50 (SD= 0.40)	Recruited through multiple UNC sources, including the Autism Research Registry, TEACCH Program, Part C early intervention providers, CIDD, and other research studies / Recruited through multiple UNC sources, including the Autism Research Registry, TEACCH Program, Part C early intervention providers, CIDD, and other research studies.	FITT	The RAND-36 (SF-36)	High risk of bias
Ilg et al. [43]	2018	France	Non-randomized interventional study	16 caregivers (Pre-intervention) / 16 caregivers (Post-intervention)	Mean: 35.00 (SD= 7.20) / Mean: 35.00 (SD= 7.20)	Mean: 3.80 (SD= 0.79) / Mean: 3.80 (SD= 0.79)	Recruited from the child psychiatry division of the Health Center at Rouffach (France) / Recruited from the child psychiatry division of the Health Center at Rouffach (France).	Parents in action: an ABC of children with autism behaviors	The Beach Center Family QOL Scale	High risk of bias
Niinomi et al. [44]	2016	Japan	Non-randomized interventional study	24 caregivers (Pre-intervention) / 24 caregivers (Post-intervention)	Mean: 39.50 (SD= 4.50) / Mean: 39.50 (SD= 4.50)	Mean: 7.00 (SD= 2.80) / Mean: 7.00 (SD= 2.80)	Recruited from public autism-related facilities in Aichi Prefecture (Japan) / Recruited from public autism-related facilities in Aichi Prefecture (Japan).	The Skippu-Mama program	WHOQOL 26	High risk of bias
Chiang et al. [45]	2014	USA	Non-randomized interventional study	9 caregivers (Pre-intervention) / 9 caregivers (Post-intervention)	NA / NA	Range: 3-11 / Range: 3-11	Recruited through a local community center in New York / Recruited through a local community center in New York.	The parent education program	WHOQOL 26	High risk of bias
Roberts et al. [46]	2011	Australia	Non-randomized interventional study	29 caregivers (Pre-intervention) / 29 caregivers (Post-intervention)	NA / NA	Pre-school age / Pre-school age	Recruited from the Children’s Hospital at Westmead and Autism Spectrum Australia / Recruited from the Children’s Hospital at Westmead and Autism Spectrum Australia.	The centre-based Building Blocks programs	The Beach Center Family QOL Scale	High risk of bias
Shu et al. [47]	2005	Taiwan	Non-randomized interventional study	8 caregivers (Pre-intervention) / 8 caregivers (Post-intervention)	Mean: 41.00 / Mean: 41.00	NA / NA	Recruited through the Society of Autism in southern Taiwan / Recruited through the Society of Autism in southern Taiwan.	Support group programme	WHOQOL-BREF (Taiwan version)	High risk of bias

Effects of RC-Containing Programs

Participants: A total of 1,030 participants were included in the five studies. The mean age of the children with ASD ranged from 5.0 to 16.2 years. The mean age of the caregivers of these children ranged from 40.0 to 48.9 years. However, the ages of the caregivers in the studies by Eskow (2019) and Eskow (2015) were not provided [34,36].

Interventions: RC-containing programs included "Autism on the Seas intervention," "Medicaid Home and Community-Based Services waiver programs," "Autism Specific Early Learning and Care Centers (ASELCCs)," and "Medicaid Home and Community-Based Services (HCBS) waiver programs." No program was combined with PT programs.

Controls: A total of 627 controls were included in the five studies. The controls in four studies by Eskow (2019, 2015, 2011) and Due (2018) were caregivers of children with ASD who had minimal or no access to RC-containing programs [34-37]. The controls in the study by SooHoo (2019) were the same participants before they received the RC-containing programs [26].

Outcomes: Outcome measurement involved three scales: "Beach Center Family Quality of Life Scale," "Quality of Life Autism (QoL-A) (parent version)," and "Family Quality of Life Scale (FQoL)," which were incorporated as one section of the MASS-R.

Type of studies: All five studies were non-randomized interventional studies. Of these, one study used a pre-post design, and four studies used a post-only design.

Quality assessment: RoBANS-2 is shown in Table 4. All five studies were scored as “High” in terms of the risk of bias. Among the eight domains of bias assessment (RoBANS-2), “Blinding of assessors,” “Comparability of the target group,” and “Confounders” domains had five, two, and one studies, respectively, rated as “High.” In addition, the “Incomplete outcome data” domain had one study rated as “Unclear.”

**Table 4:** Quality assessment of the included studies on RC-containing programs (RoBANS-2) RC: respite care; RoBANS-2: The Revised Risk of Bias Assessment Tool for Nonrandomized Studies Source: [30]

Author / Year	Comparability of the Target Group	Target Group Selection	Confounders	Measurement of Intervention/Exposure	Blinding of Assessors	Outcome Assessment	Incomplete Outcome Data	Selective Outcome Reporting	Overall Risk of Bias
SooHoo 2019 [26]	Low	Low	Low	Low	High	Low	Unclear	Low	High
Eskow et al. 2019 [34]	Low	Low	Low	Low	High	Low	Low	Low	High
Due et al. 2018 [35]	Low	Low	High	High	High	Low	Low	Low	High
Eskow et al. 2015 [36]	Low	Low	Low	Low	High	Low	Low	Low	High
Eskow et al. 2011 [37]	High	Low	High	Low	High	Low	Low	Low	High

Effects of PT Programs

Participants: A total of 281 participants were included in the 10 studies. The average age of children with ASD in these studies ranged from 2.4 to 11.3 years. However, in the study by Roberts et al. [46], ages were not specified and were only indicated as "Pre-school age." Additionally, the mean age of the caregivers of these children ranged from 35.0 to 43.7 years.

Interventions: PT was implemented through various programs. These programs included "The center-based Building Blocks programs," "Family Implemented TEACCH for Toddlers (FITT)," "The parent-training program named 'Parents in action: an ABC of children with autism behaviors'," "The Skippu-Mama program," "The parent education program," "Support group program," "Ingersoll’s parent training protocol," "The CARES program," "The Intervention: IPAT Training Activity Using the IPAT Module," and "a culturally adapted, group-based parent coaching program via telehealth." In addition, none of these PT programs combined RC-containing programs or other PT programs.

Controls: A total of 281 controls were included in the 10 studies. A pre-post research design was employed in all studies; therefore, no control groups were established. The controls in all studies were the same participants before they received the PT programs.

Outcomes: The measurement of caregiver's QoL outcomes included the utilization of "World Health Organization-Quality of Life 26 (WHOQoL 26)" in six studies, "The Beach Center Family Quality of Life Scale" in two studies, "The RAND-36" in one study, and "Quality of Life Enjoyment Satisfaction Questionnaire-Short Form (QLESQ-SF)" in one study.

Type of studies: The research design for all studies was exclusively pre-post.

Quality assessment: RoBANS-2 is shown in Table 5. All 10 studies were scored as “High” in terms of the risk of bias. Among the eight domains of bias assessment (RoBANS-2), the “Blinding of assessors” domain was rated as “High” in all 10 studies. Eight studies were rated as “Unclear” in the “Incomplete outcome data” domain.

**Table 5:** Quality assessment of the included studies on PT programs (RoBANS-2) PT: parent training; RoBANS-2: The Revised Risk of Bias Assessment Tool for Nonrandomized Studies Source: [30]

Author / Year	Comparability of the Target Group	Target Group Selection	Confounders	Measurement of Intervention/Exposure	Blinding of Assessors	Outcome Assessment	Incomplete Outcome Data	Selective Outcome Reporting	Overall Risk of Bias
Qu et al. 2024 [38]	Low	Low	Low	Low	High	Low	Low	Low	High
Mavroeidi et al. 2024 [39]	Low	Low	Low	Low	High	Low	Unclear	Low	High
Mills et al. 2021 [40]	Low	Low	Low	Low	High	Low	Unclear	Low	High
Akhani et al. 2021 [41]	Low	Low	Low	Low	High	Low	Unclear	Low	High
Turner-Brown et al. 2019 [42]	Low	Low	Low	Low	High	Low	Low	Low	High
Ilg et al. 2018 [43]	Low	Low	Low	Low	High	Low	Unclear	Low	High
Niinomi et al. 2016 [44]	Low	Low	Low	Low	High	Low	Unclear	Low	High
Chiang et al. 2014 [45]	Low	Low	Low	Low	High	Low	Unclear	Low	High
Roberts et al. 2011 [46]	Low	Low	Low	Low	High	Low	Unclear	Low	High
Shu et al. 2005 [47]	Low	Low	Low	Low	High	Low	Unclear	Low	High

Meta-Analysis

**Figure 3:**
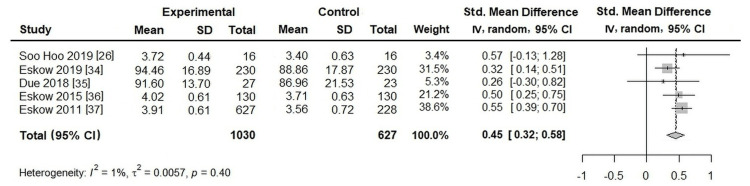
Effects of RC-containing programs on caregiver QoL Sources: [26,34-37]

Effects of RC-containing programs: Five studies were included to evaluate the effects of RC-containing programs. As shown in Figure 3, the meta-analysis demonstrated a moderate and statistically significant overall effect (SMD = 0.45; 95% CI: 0.32-0.58). Heterogeneity was low (I² = 1%, τ² = 0.0057, p = 0.40), indicating that effect sizes were largely consistent across studies. Because only five studies were available, publication bias was not assessed.

**Figure 4:**
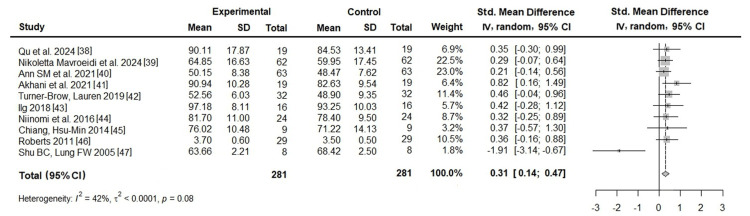
Effects of PT programs on caregiver QoL Sources: [38-47]

Effects of PT programs: Ten studies were included to evaluate the effects of the PT programs. As indicated in Figure 4, the meta-analysis revealed a significant effect of PT programs, with an SMD of 0.31 (95% CI: 0.14-0.47). However, heterogeneity was high (I² = 42%, τ² < 0.0001, p = 0.08). Publication bias was not assessed because of the limited sample size (n=10).

Discussion

In the present systematic review, RC-containing programs and PT programs for caregivers of children with ASD were found to be significantly effective in improving their QoL. However, all studies included in this review were considered to have a high risk of bias, demanding cautious interpretation.

RC-containing programs have been suggested to improve the QoL of caregivers of children with ASD. All professionals supporting children with ASD and their parents are expected to recognize RC as a caregiver’s right and plan and manage RC as part of family support programs. For example, the necessity and priority of RC should be determined through family interviews conducted in all settings where the family and professional staff interact, such as during the child's outpatient visits, home-visit nursing, or the utilization of daycare or residential services. During these processes, family assessments should be conducted based on the primary caregiver's burnout symptoms, sleep patterns, fatigue, health-compromising behaviors, and daily living conditions. According to the family's needs, effective RC can be proposed to alleviate acute or chronic stress and improve parental QoL. Because RC comes in various forms, it is important to plan and manage the optimal RC, such as in-home or facility-based care, temporary or overnight stays, and home helpers, to support the children or parents in each family.

Receiving RC reduces mental stress for caregivers and increases positive experiences in daily life [28,48]. This is expected to improve the quality of family relationships, such as marital and parent-child relationships, and ultimately improve parental QoL [49]. Meanwhile, several studies have suggested that PT fosters parenting self-efficacy, enabling parents to learn effective caregiving strategies, thereby reducing stress and enhancing psychological well-being [50,51]. Boosting parental confidence and satisfaction through these programs may improve parent-child interactions and family relationships, ultimately contributing to higher family QoL [51]. Moreover, PT helps parents gain a deeper understanding of their children with ASD, reduces emotional stress, and potentially improves parental QoL [41,46]. In other words, RC contributes to reducing physical and mental stress and improving the short-term QoL of families through temporary or short breaks. However, PT has the potential to improve family QoL through long-term improvements in parenting skills.

The common element between RC and PT is that families begin to interact with society. Increased social participation and community engagement reduce the likelihood that parents and children become isolated, facilitate access to social support and understanding, and ultimately improve family QoL [52]. According to previous studies targeting families with disabilities, families that received comprehensive RC, recreation, counseling, and social support coordination reported a significant improvement in family QoL compared to groups that received only RC [53]. In other words, it is suggested that combining various support activities, rather than simply providing rest, can lead to improved family QoL. Therefore, comprehensive family support programs that combine multifaceted support tailored to caregivers' needs can be effective for various aspects of family life, including family QoL, family interaction, parent-child relationships, emotional stability, and physical well-being.

Although we conducted the present systematic review and meta-analysis in accordance with the PRISMA 2020 guidelines, the non-randomized interventional study contained an inherent risk of bias as revealed by RoBANS-2. Another key limitation of the present systematic review was the heterogeneity of the intervention. In real-world practice, RC-containing programs for children with ASD typically involve multi-component approaches, making it difficult to strictly evaluate the sole effects of RC. Indeed, RC-containing programs involve several support programs implemented in close collaboration with families [54]. This limits the ability of the present systematic review to draw definitive conclusions regarding the effects of RC-containing programs. Future research should precisely define the components and protocols of RC-containing programs to evaluate their true effects. Moreover, RCTs and high-quality non-randomized interventional studies are required to disentangle the contributions and potential interactions of RC and combined support programs within real-world RC-containing programs.

## Conclusions

The present systematic review and meta-analysis included a total of five studies that investigated the effects of RC-containing programs and 10 studies of PT programs, a well-recognized family support approach, on the QoL of caregivers of children with ASD. The results demonstrated that RC-containing programs are beneficial for improving the QoL of caregivers of children with ASD, and their effects are comparable to those of PT programs. However, all included studies in the present systematic review had a high risk of bias, especially since RC-containing programs were inherently multi-component and heterogeneous interventions. Further RCTs and high-quality non-randomized interventional studies would be required to clarify the contributions and potential interactions of RC and combined support programs. Professionals supporting children with ASD need to be aware of the significance of RC and RC-containing programs.
